# Effects of Dietary Protein Levels on Meat Quality, Serum Antioxidant Capacity, and Intestinal Microorganisms in Ningxiang Pigs

**DOI:** 10.3390/antiox14040415

**Published:** 2025-03-30

**Authors:** Shanghang Liu, Kai Yang, Jie Yin, Jiashun Chen, Qian Jiang, Jing Wang, Bie Tan, Xiaokang Ma, Juan Liu

**Affiliations:** Key Laboratory of Hunan Province for the Products Quality Regulation of Livestock and Poultry, College of Animal Science and Technology, Hunan Agricultural University, Changsha 410128, China; y1ko@stu.hunau.edu.cn (S.L.);

**Keywords:** Ningxiang pigs, serum antioxidant, protein nutrition, growth performance, meat quality, intestinal health

## Abstract

This study aimed to evaluate the impact of five different dietary protein levels on meat quality, serum antioxidant capacity, and intestinal microorganisms of Ningxiang pigs, thus providing new insights into their nutritional needs. One hundred and twenty-five healthy Ningxiang barrows with an average body weight of 53.19 ± 2.12 kg were randomly divided into five groups with five replicates and five pigs per replicate. The diet was formulated using corn, soybean meal, and rice bran meal as raw materials based on net energy. Following the nutritional requirements outlined in the Nutrient Requirements of Swine in China (2020), diets with five different protein levels (crude protein: 11.09%, 12.09%, 13.09%, 14.09%, 15.09%) were formulated. The amino acid levels of lysine, threonine, tryptophan, methionine, valine, isoleucine, and other amino acids were standardized to meet the recommended values, as were other essential amino acids. The experiment lasted for 62 days. The results indicated a linear decrease in the redness and yellowness values of the *Longissimus dorsi* muscle with increased dietary protein, alongside a quadratic decrease in intramuscular fat (*p* < 0.01). Notably, muscles from pigs fed with 13.09%, 14.09%, and 15.09% protein diets exhibited significantly lower redness and yellowness than those on a 12.09% protein diet (*p* < 0.05). Additionally, higher dietary protein levels linearly enhanced the presence of specific fatty acids (C17:0, C17:1, C18:3n3, and C18:3n6) and certain amino acids in the *Longissimus dorsi* muscle, following a quadratic trend (*p* < 0.01). The serum GSH-Px content increased linearly with greater dietary protein levels (*p* < 0.05). Significant variations in cecal and colonic metabolites were observed across different protein levels, affecting the contents of putrescine, cadaverine, spermine, spermidine, and short-chain fatty acids (*p* < 0.05). Additionally, the increase in dietary protein levels correlates with the growth performance and amino acid profile of the *Longissimus dorsi* muscle in Ningxiang pigs, presenting a quadratic relationship. Concurrently, the serum antioxidant capacity and cecal bioamine content demonstrate a linear increase. Despite a balanced inclusion of six essential amino acids, both excessively high and low protein levels adversely affect growth and intestinal health. Notably, dietary protein levels of 12.09% and 13.09% yield the optimal growth performance under the specified experimental conditions.

## 1. Introduction

Protein scarcity is a pressing global issue, worsened in densely populated regions with limited resources. Numerous sectors, including healthcare, nutrition, and aquaculture, face distinct challenges due to varying protein needs. It is crucial to strategically reduce protein usage across various fields or seek alternative resources, which is essential to alleviate the protein shortage. Expensive and limited protein additives cannot sustainably satisfy the growing demands of animal agriculture, as evidenced by the global deficiency in feed protein, which impacts animal production [1]. In this context, reducing feed protein levels can help save on feed costs. Research by Li et al. [2] revealed that pigs on a 13% protein diet exhibited enhanced MyHC-1 and MyHC-lla muscle fiber expression, softer meat, and increased intramuscular fat (IMF) along with monounsaturated fatty acids (MUFAs), contributing to a better meat color and flavor. Wang et al. [3] reported that in summer, lowering dietary crude protein levels from 12% to 10% while supplementing with restricted amino acids had no negative effects on growth performance indices such as the average daily gain (ADG), average daily feed intake (ADFI), and feed-to-gain ratio (F:G) compared to a normal protein diet.. Conversely, Morales et al. [4] demonstrated that increasing the protein level to 21.6% significantly increased the improved ADG and G:F to counteract reduced amino acid intake caused by heat stress, despite a possible rise in body temperature. However, excessive protein intake should be avoided due to its potential negative effects on kidney function and overall health, as too much protein can strain the body [5,6]. By selectively adjusting feeding regimens to include high-protein diets, it may be possible to optimize production without compromising health.

Ningxiang pigs are native to Liushahe, Caochong, and other areas within Ningxiang County, Hunan Province, boasting a history of over 1000 years in China. These pigs display an exceptional ability for fat deposition—nearly double that of their leaner counterparts, achieving a rate of 42.3% compared to 21.9% [7]. Distinguished by their early maturity and considerable fat accumulation capabilities, Ningxiang pigs are recognized as the quintessential semi-fatty breed among various indigenous Chinese pigs bred for both meat and fat production. Given their growth characteristics, Ningxiang pigs require a specialized feeding approach; high-energy, high-protein diets are less effective since they do not improve the feed conversion efficiency and may unnecessarily increase fat storage. On the other hand, an insufficient protein intake can adversely impact growth and lead to excessive fat buildup. Compared to hybrid breeds such as Changbai, Large White, and Duroc, the specific protein and amino acid needs of local Chinese pig breeds like Ningxiang remain inadequately studied. Therefore, developing precise feeding standards is vital to address the growing consumer demand for high-quality pork quality in China. In recent years, researchers have sought to reduce the nutrient levels for Ningxiang pigs during the fattening phase, likely considering their carcass traits and the need to conserve feed resources. In light of the dual pressures from feed resource scarcity, environmental pollution in animal husbandry, and market demand for high-quality animal products, it is crucial to appropriately adjust the diet nutrient level combination and explore its effects on growth performance and carcass traits for the sustainable development of Ningxiang pig germplasm resources.

The objective of this research was to thoroughly assess how five different protein levels impact the growth performance, meat quality, and intestinal metabolites in Ningxiang finishing pigs. This study aims to create a solid experimental foundation to guide the nutritional strategies tailored to the unique dietary requirements of Ningxiang pigs.

## 2. Material and Methods

### 2.1. Animal Treatment and Experimental Design

One hundred and twenty-five healthy Ningxiang barrows with an average body weight of 53.19 ± 2.12 kg were randomly divided into five groups with five replicates and five pigs per replicate. The diet was formulated using corn, soybean meal, and rice bran meal as raw materials based on net energy. Following the nutritional requirements outlined in Nutrient Requirements of Swine in China (2020), diets with five different protein levels (crude protein: 11.09%, 12.09%, 13.09%, 14.09%, 15.09%) were formulated. The amino acid levels of lysine, threonine, tryptophan, methionine, valine, isoleucine, and other amino acids were standardized to meet the recommended values, as were other essential amino acids. Energy levels, trace elements, minerals, and other dietary components met the recommended values and maintained consistency (Table 1). The fatty acid content of the experimental diets was evaluated (Table 2).

The protocol of this study was approved by the Institution Animal Care and Use Committee of the College of Animal Science and Technology (ACC2023040, 26 May 2023), Hunan Agricultural University (Changsha, China), and an animal feeding experiment was conducted at the Ningxiang Animal Testing Base in Hunan Province, lasting 62 days. Pigs were raised in a fully leaky seam floor piggery, with free access to food and water, and managed according to the routine procedures for deinsectization and immunization, and regular cleaning and disinfection of the piggery. The ambient temperature in the housing was automatically regulated by the thermostat, and the windows were opened periodically for ventilation at regular intervals. During the experiment, the body weight and feed intake were recorded. These data were used to calculate the average daily gain (ADG), average daily feed intake (ADFI), and feed gain ratio (F/G). On the 62nd day of the experiment, all pigs were fasted for 12 h, and one pig was randomly selected from each pen (*n* = 5) for blood collection. Ten milliliters of blood were collected in the jugular vein of the pigs to separate the serum, which was then stored at −20 °C. After slaughter, routine samples of *Longissimus dorsi* muscle were collected and frozen at −20 °C for subsequent meat quality analysis. Approximately 10 g of cecal chyme samples was collected and stored in a −80 °C refrigerator for the analysis of volatile fatty acids and biogenic amines and subsequent microbial composition analysis.

### 2.2. Direction Indicators

#### 2.2.1. Growth Performance

The average daily gain (ADG), average daily feed intake (ADFI), and feed to gain (F/G) were calculated based on BW measured weekly.

#### 2.2.2. Assessment of Carcass Characteristics

After the feeding experiment and fasting for 12 h, slaughter was carried out. The carcass weight was recorded after slaughter, and the slaughter rate was calculated. After the carcass was divided, a tape measure was used to measure the straight length of the carcass (the length from the leading edge of the pubic symphysis to the depression of the first cervical vertebra) and the oblique length of the carcass (the length from the leading edge of the pubic symphysis to the inner edge of the first rib to the sternum). Backfat thickness and eye muscle area were measured with vernier calipers, in which the subcutaneous fat thickness at the thickest part of the shoulder (point A), the last rib (point B), and the lumbosacrum junction (point C) of the carcass was measured to calculate the mean backfat thickness. To calculate the eye muscle area, we measured the maximum width and length of the eye muscle. The lean meat was weighed, and the lean meat percentage was calculated.

#### 2.2.3. Meat Quality

The pH of the *Longissimus dorsi* muscle was measured 45 min and 24 h after slaughter, respectively. In short, we measured the pH at the same position at the *Longissimus dorsi* muscle using a digital pH meter (HI99161, HANNA, Padova, Italy) and adjusted the pH meter before use using a standard liquid (pH = 4.01, 6.86 and 9.18).

Meat color traits (lightness L*, redness a*, and yellowness b*) were measured by the reflectance spectrophotometer Minolta CR-410 (Kinica Minolta Sensing Inc., Osaka, Japan).

Drip loss of the *Longissimus dorsi* muscle was measured according to the method of Honikel et al. [8]. In short, we weighed about 50 g of fresh *Longissimus dorsi* muscle sample, placed it in a whirl-Pak bag, hung it in a 4 °C cooler for 48 h, and re-weighed the sample. The result was expressed as a percentage of weight loss.

The water-holding capacity was tested using the pressing loss. According to Zhang et al.’s [9] method, briefly, a sample of *Longissimus dorsi* muscle about 1 cm thick was prepared and sampled with a 2.523 cm sampler. The weight was taken by a scale and the meat sample was wrapped with two sheets of gauze; then, 18 pieces of filter paper were placed on the pressure instrument. We set the pressure to 35 kg and held that for 5 min, removed the meat sample, and weighed again. Water-holding capacity: WHC = (Initial weight − Final weight)/Initial weight × 100%.

The shear force was measured by a shear instrument (Model 1011, Instron instrument, Norwood, MA, USA). The samples of *Longissimus dorsi* muscle were heated in a 72 °C water bath to the central temperature of 70 °C, then removed immediately. After cooling to room temperature with running water, the meat samples were drilled with a circular sampler with a diameter of 1.27 cm in the direction parallel to the muscle fibers. Then, the sample was repeated 3 times, the shear force value of the meat sample was recorded 3 times, and the average value was calculated. 

The analysis of intramuscular fat content was measured according to the Soxhlet method. We dried 20 g of fresh *Longissimus dorsi* muscle in a vacuum freeze-drier and pulverized the sample. Then, one gram of homogenized power was placed in a thimble (22 × 28 mm, i.d. Foss North America, Eden Prairie, MN, USA), fitted with metal adaptors, and loaded into an Automated Soxtherm Fat Extraction System (Gerhardt, Königswinter, Germany). After extraction, the extract was dried in an oven at 104 °C and cooled in a desiccator to determine fat gravimetrically.

#### 2.2.4. Determination of Fatty Acid Profiles in *Longissimus dorsi* Muscle

After the freeze-dried pork sample was ground, 0.5 g of freeze-dried sample was weighed, benzene-petroleum ether (1:1) was added, and the fat was extracted for 24 h in a closed manner. Then, 4 mL of potassium hydroxide methanol (KOH, 0.4 mol/L) was added to it for methyl esterification. After completion, we added ultrapure water to the layer, took the upper solution, and added anhydrous sodium sulfate to absorb water in the sample. We took out 200 μL of the sample, added 800 μL of n-hexane, diluted it, and passed the solution through a 0.22 um filter. Fatty acids were determined by gas chromatography (Agilent 6890, Boston, MA, USA) and the results were expressed as a percentage of total fatty acids.

#### 2.2.5. Determination of Amino Acid Profiles in *Longissimus dorsi* Muscle

The amino acid composition of *Longissimus dorsi* muscle samples was determined by ultra-high performance liquid chromatography (UHPLC-HRMS). The preparation method of the samples was as follows: Firstly, a certain amount of sample (about 50 mg) was weighed and homogenized, 1000 μL of 50% methanol was added, and the sample was ultrasonically extracted for 30 min and centrifuged at 12,000 rpm for 10 min at 4 °C. We took 10 μL of supernatant and added 10 μL of ultra-pure water, 5 μL of internal standard, and 40 μL of isopropyl alcohol (0.1% formic acid) in a 1.5 mL centrifuge tube. Then, we centrifuged at 12,000 rpm and 4 °C for 10 min. Finally, 10 μL of the supernatant was placed in a 1.5 mL centrifuge tube, and 70 μL of boric acid buffer salt and 20 μL of AccQ Tag (Kairos) derivative reagent were added. Chromatographic separation was performed by Waters ultra-high-performance liquid chromatography.

#### 2.2.6. Blood Antioxidant Capacity

According to the manufacturer’s instructions (Nanjing Jiancheng Bioengineering Institute, China), the total antioxidant capacity (T-AOC) and contents of superoxide dismutase (SOD), glutathione peroxidase (GSH-Px), and malondialdehyde (MDA) in the serum of growing pigs were measured with a spectrophotometer (cold light sfz160017568, Shanghai, China).

#### 2.2.7. Cecal Biogenic Amine Content

Cecal biogenic amines were determined via high-performance liquid chromatography. About 0.5 mL supernatant of the mixed liquid sample was accurately removed into a 15 mL centrifuge tube, 1 mL of NAOH solution, and 20 μL of benzoyl chloride were added, and the reaction was carried out in a liquid mixer for 30 s. The reaction was carried out in a 37-degree water bath for 20 min, during which the mixture was rotated for 30 s every 5 min. After the derivation, 2 mL of saturated sodium chloride solution and 2 mL of anhydrous ether were added, mixed with oscillations, and left to stand; then, the supernatant liquid nitrogen was removed and blown to dry, and 0.5 mL of methanol (HPLC grade) was added to dissolve the product, before it was passed through a 0.22 μm filter membrane. A Waters ALLiance high-performance liquid chromatograph (e2695) was used for determination. The contents of various biogenic amines were calculated with a standard curve using the external standard method.

#### 2.2.8. Short-Chain Fatty Acid Analysis

Short-chain fatty acids in colonic chymus were determined by gas chromatography. We accurately weighed 60 mg of fresh samples and recorded the mass of each sample, then placed them in 2 mL homogenizing tubes, added 1.5 mL of ultra-pure water, homogenized at 4000 r for 30 s, repeated twice, left overnight at 4 °C, centrifuged at 10,000 r/min for 10 min, transferred the supernatant to a 2 mL centrifuge tube, added v:v = 4:1 (400 μL of supernatant + 100 μL of 25% metaphosphoric acid) in a 2 mL centrifuge tube, mixed well, left to stand at room temperature for 3–4 h, centrifuged with a 45 um microporous filter membrane (nylon system), and added to a machine bottle (more than 600 μL) for testing.

#### 2.2.9. Microbiota Analysis by 16S RNA

The cecal chyme samples were sequenced using 16s rRNA, including DNA extraction and detection, PCR amplification, purification, library construction, library quality inspection and sequencing, and data analysis, which were all performed at Shanghai Meiji Biotechnology Co., Ltd. (Shanghai, China). Total genome DNA was extracted from cecal chyme samples of Ningxiang pigs using the QIAamp Fast DNA Stool mini kit (Qiagen, Hilden, Germany) and checked with 1% agarose gel. The DNA concentration and purity were determined with a Nano Drop 2000 UV-vis spectrophotometer (Thermo Fisher Scientific, Wilmington, NV, USA). Target sequences such as microbial ribosomal RNA, which can reflect the composition and diversity of bacterial flora, were taken as targets, corresponding primers were designed according to the conserved regions in the sequences, specific barcode sequences were added to the samples, and then PCR amplification was performed on the variable region (single or multiple consecutive) of rRNA genes or specific gene fragments. Q5 high-fidelity DNA polymerase from NEB was used for PCR amplification, and the number of amplification cycles was strictly controlled to keep the number of cycles as low as possible and ensure the same amplification conditions for the same batch of samples. The PCR products were detected by 2% agarose gel electrophoresis, and the target fragments were gelled and recovered. According to the preliminary quantitative results of electrophoresis, the recovered product was then subjected to a fluorescence assay by using the Quant-iT PicoGreen dsDNA Assay Kit, with a microplate reader as the quantitative instrument (BioTek, Winooski, VT, USA, FLx800). Illumina’s TruSeq Nano DNA LT Library Prep Kit was used to prepare sequencing libraries. The raw 16S rRNA gene-sequencing reads were demultiplexed, quality-filtered, and merged according to previous studies [10,11]. The a-diversity of cecal microbiota was evaluated with the Ace, Chao richness, Shannon, and Simpson indexes. B-diversity was evaluated using principal component analysis (PCoA) based on the Euclid distance. OTUs representing < 0.005% of the population were removed and taxonomy was assigned using the RDP classifier. The relative abundance of each OTU was counted at different taxonomic levels. Then, bioinformatics analysis was mainly performed using QIIME (v1.7.0) and R packages (v3.2.0). The OTU table in QIIME was used to calculate the OTU level, and β-diversity was assessed by principal coordinate analysis (PCoA). The cluster analysis and significant differences between samples were tested by ANOSIM.

### 2.3. Statistical Analysis

All data were analyzed by one-way analysis of variance (ANOVA) using the general linear model (GLM) procedures (SAS Institute Inc., Cary, NC, USA) in a randomized block design. An orthogonal polynomial comparison method was used to determine the linear and quadratic effects of the dietary protein level on various traits. The pen was the experimental unit for the analyses of the performance data, and the pig was the experimental unit for the analyses of other data in the present study. All results were expressed as the mean and SEM. Differences were considered to be significant at *p* < 0.05.

## 3. Results

Table 3 illustrates that when dietary protein was either decreased to 11.09% or increased to 15.09%, both the final body weight and average daily gain significantly diminished compared to the 12.09% protein group *(p* < 0.05). Additionally, an increase in dietary protein resulted in a quadratic reduction in both final weight and average daily gain, accompanied by a quadratic rise in the feed-to-gain ratio (*p* < 0.01).

Table 4 demonstrated that the redness (a*) and yellowness (b*) values in the *Longissimus dorsi* muscle were significantly reduced for protein levels of 13.09%, 14.09%, and 15.09% compared to the baseline 12.09% protein level (*p* < 0.05). The intramuscular fat contents for protein levels of 11.09% and 15.09% were significantly increased compared with that for a protein level of 13.09%. Additionally, a linear increase in dietary protein resulted in a linear decline in both the redness and yellowness of the muscle, while the content of intramuscular fat exhibited a quadratic reduction (*p* < 0.01).

Table 5 indicates that the levels of C17:0, C17:1, and C18:3n3 fatty acids in the *Longissimus dorsi* muscle were significantly higher in pigs fed a 15.09% protein diet compared to those on a 12.09% protein diet (*p* < 0.05). Furthermore, as dietary protein levels increased, the concentrations of C17:0, C17:1, C18:3n3, and C18:3n6 fatty acids in the muscle tissue showed a consistent linear increase (*p* < 0.01).

As shown in Table 6, the contents of glycine (Gly), alanine (Ala), leucine (Leu), tyrosine (Tyr), phenylalanine (Phe), arginine (Arg), proline (Pro), non-essential amino acid (NEAA), and total amino acid (TAA) in *Longissimus dorsi* muscle were significantly lower in pigs fed 11.09% and 15.09% protein levels compared to those on 13.09% and 14.09% protein levels (*p* < 0.05). The contents of threonine (Thr), glutamic acid (Glu), valine (Val), histidine (His), and essential amino acid (EAA) in *Longissimus dorsi* muscle were significantly lower in pigs fed a 11.09% protein level compared to those on 13.09% and 14.09% protein levels (*p* < 0.05). In addition, with the increase in dietary protein level, the contents of threonine, glutamic acid, glycine, alanine, valine, leucine, tyrosine, phenylalanine, histidine, arginine, proline, essential amino acid, non-essential amino acid, and total amino acid in *Longissimus dorsi* muscle exhibited a quadratic curve change (*p* < 0.01).

According to Table 7, the GSH-Px in the 11.09% dietary protein level group was significantly lower than that in the other four groups, and the serum GSH-Px content was linearly increased with the increase in dietary protein level (*p* < 0.05). There were no significant differences in serum SOD, MDA, and T-AOC.

As shown in Table 8, compared with the 12.09% protein level group, the contents of cademine and spermidine in cecal chyme in the 11.09% protein level group were significantly increased, and spermidine content was significantly decreased (*p* < 0.05). Putrescine, spermidine, and cadaverine increased linearly with increasing dietary protein levels (*p* < 0.05). On the other hand, spermine was lower, with dietary protein levels greater than 11.09% (*p* < 0.05).

As shown in Table 9, for isobutyric acid, the 14.09% protein group was higher than the 11.09, 12.09, and 13.09% groups (*p* < 0.05). For butyric acid, the 14.09% protein group was higher than the 12.09 and 13.09% groups (*p* < 0.05). In isovaleric acid, the 14.09% group was higher than the other treatments (*p* < 0.05).

**Figure 1:**
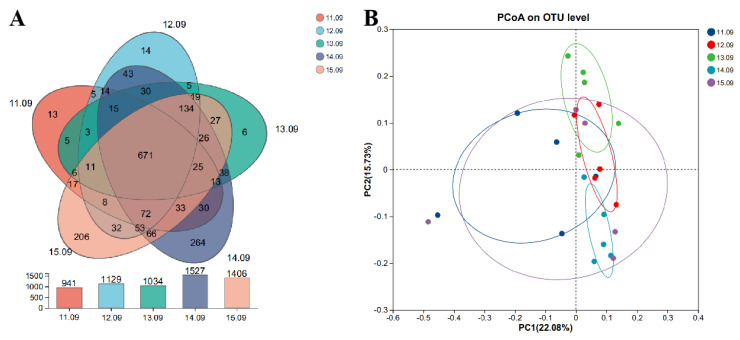
(**A**) Effects of different dietary protein levels on OTU of cecal microbiota in Ningxiang pigs at the fattening stage. (**B**) Principal coordinate analysis (PCoA) of microbial composition in the feces of growing pigs (based on the Bray–Curtis distance).

The Venn diagram in Figure 1 shows the effects of different dietary protein levels on the OTUs of cecal microbiota in Ningxiang pigs at the fattening stage. From low to high dietary protein levels, 941, 1129, 1034, 1527, and 1406 OTUs were detected, respectively, and a total of 671 OTUs were detected in the five groups (Figure 1A). The β-diversity of cecum flora in each group was characterized by principal axis analysis (Figure 1B). The group with a dietary protein level of 13.09% showed a significant separation from the other four groups, indicating a significant difference in microbial composition.

**Figure 2:**
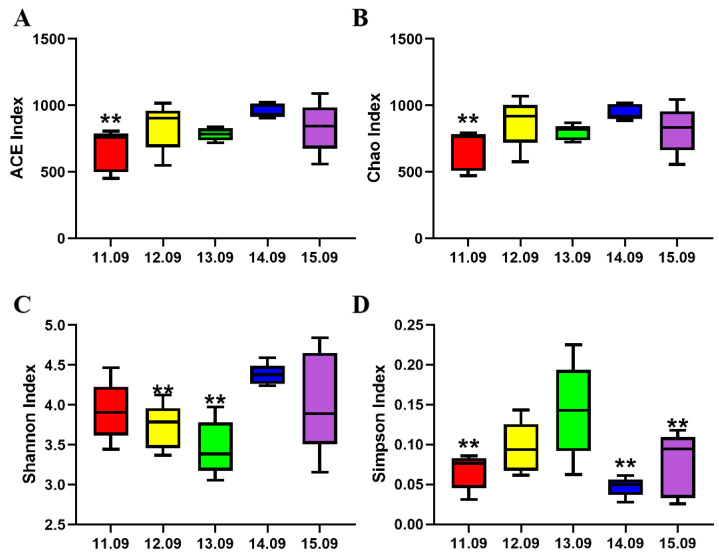
Effects of different dietary protein levels on α-diversity of cecal microbiota at the fattening stage of Ningxiang pigs. (**A**) Ace index, (**B**) Chao index, (**C**) Shannon index, (**D**) Simpson index. An individual pig was regarded as the experimental unit (*n* = 5). ** represent significant difference, *p* < 0.05.

The α-diversity of cecum microbiota is shown in Figure 2, and the Ace index, Chao index, and Simpson index of the 11.09% dietary protein level group were significantly reduced. The Shannon indexes of the 12.09% dietary protein level group and 13.09% dietary protein level group were significantly decreased.

**Figure 3:**
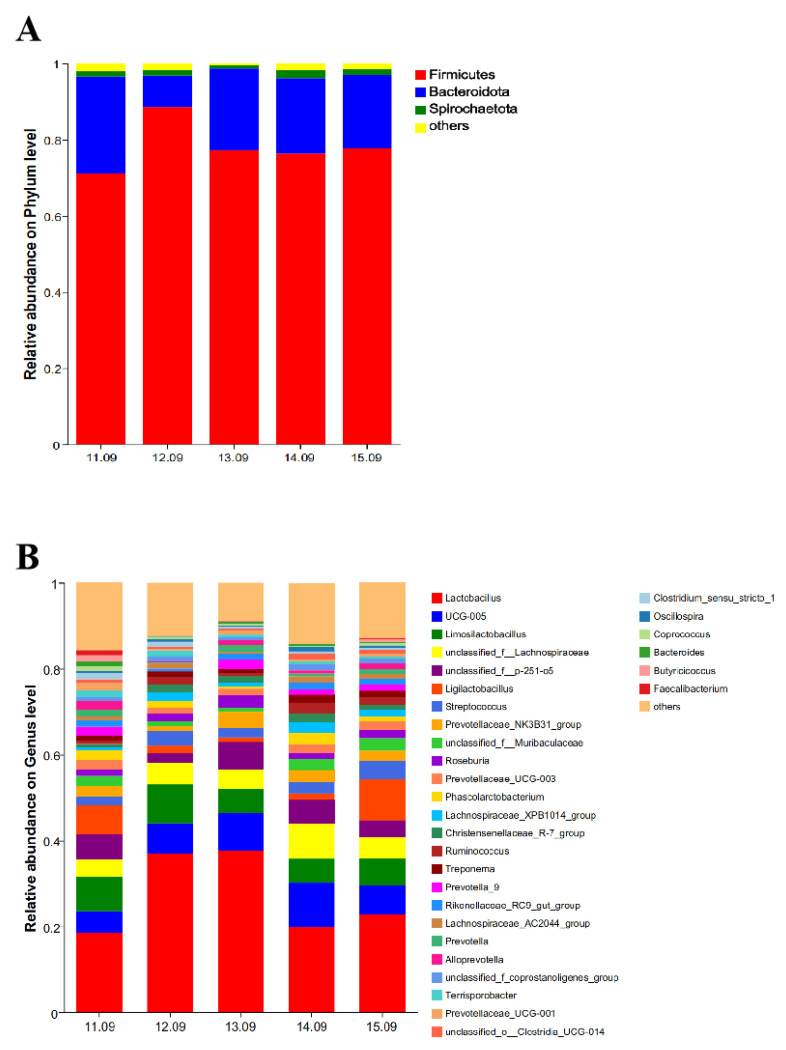
Relative abundance of cecal bacteria at the phyum (**A**) and genus (**B**) levels. The individual pig was regarded as the experimental unit (*n* = 5).

As shown in Figure 3, at the phylum level, three dominant phyla were detected in the five groups. Firmicutes, Bacteroidota, and Spirochaetota were the dominant phyla, accounting for 71.11, 25.43, and 1.35%, respectively, in the 11.09% dietary protein level group; 88.55, 8.26, and 1.52%, respectively, in the 12.09% dietary protein level group; 77.31, 21.39, and 0.39%, respectively, in the 13.09% dietary protein level group; 76.29, 19.90, and 2.06%, respectively, in the 14.09% dietary protein level group; and 77.72, 19.27, and 1.57%, respectively, in the 15.09% dietary protein level group (Figure 3A). At the genus level, a total of 31 genera were detected in the five groups. The dominant genera in the 11.09% dietary protein level group were *Lactobacillus*, *Limosilactobacillus*, *Ligilactobacillus*, *unclassified_f__p-251-o5,* and *UCG-005*. The proportions were 18.44, 8.11, 6.68, 5.87, and 5.08%, respectively. The dominant strains in the 12.09% dietary protein level group were *Lactobacillus*, *Limosilactobacillus*, *UCG-005*, *unclassified_f__Lachnospiraceae,* and *Streptococcus*. The proportions were 36.95, 9.09, 7.00, 4.99, and 3.50%, respectively. The dominant genera in the 13.09% dietary protein level group were *Lactobacillus*, *UCG-005*, *Limosilactobacillus*, *unclassified_f__p-251-o5,* and *unclassified_f__Lachnospiraceae*. The proportions were 37.84, 8.78, 6.22, 5.63, and 4.61%, respectively. The dominant genera in the 14.09% dietary protein level group were *Lactobacillus*, *UCG-005*, *unclassified_f__Lachnospiraceae*, *Limosilactobacillus,* and *unclassified_f__p-251-o5*. The proportions were 19.84, 10.35, 8.15, 5.60, and 5.57%, respectively. The dominant genera in the 15.09% dietary protein level group were *Lactobacillus*, *Ligilactobacillus*, *UCG-005*, *Limosilactobacillus,* and *unclassified_f__Lachnospiraceae*. The proportions were 22.86, 9.61, 6.56, 6.45, and 4.98%, respectively (Figure 3B).

**Figure 4:**
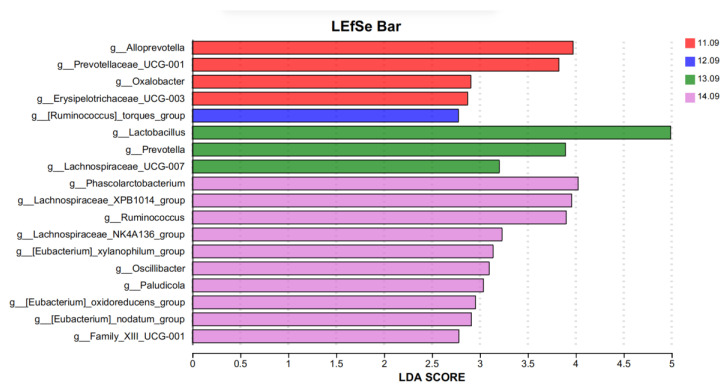
Identification of the most differentially abundant genera in cecal microbiota. The plot is generated from linear discriminant effect size (LEfSe) analysis with a CSS-normalized OTU table and displays taxa with LDA scores above 2.50 and *p*-values below 0.05.

Lefse analysis was used to analyze and identify bacteria with significant differences at the genus level in different protein levels treated in five diets (Figure 4, LDA = 2.5). Lefse analysis was used to analyze and identify bacteria with significant differences at the genus level in different protein levels treated in the five diets (Figure 4, LDA = 2.5). Four species of differential bacteria (*Alloprevotella*, *Prevotellaceae_UCG-001*, *oxalter,* and *Erysipelotrichaceae_UCG-003*) were significantly enriched at the 11.09% dietary protein level group. *Ruminococcus]_torques_group* was significantly enriched in the 12.09% dietary protein level group and three distinct strains (*Lactobacillus*, *Prevotella,* and *Lachnospiraceae_UCG-007*). Ten species of bacteria (*Phascolarctobacterium*, *Lachnospiraceae_XPB1014_group*, *Xylanophilum_group*, *Ruminococcus*, *Lachnospiraceae_NK4A136_group, Oscillibacter*, *Paludicolag*, *Oxidoreducens_group*, *Nodatum_group,* and *g__Family_XIII_UCG-001*) were significantly enriched in the 14.09% diet protein group.

## 4. Discussion

Protein plays a critical role in animal growth and metabolism. However, an excessive protein level in swine diets is correlated with a decline in [12]; conversely, judicious restriction of crude protein levels can enhance meat quality, reduce production costs, and mitigate nitrogen pollution [13]. The findings of the present study indicate that both high-protein and low-protein diets exert inhibitory effects on growth performance. This phenomenon may be attributable to fluctuations in the concentrations of certain non-restrictive amino acids, which disrupt the pre-existing amino acid balance pattern. From the specific effects of protein on pig growth and metabolism, it can be inferred that protein is the main component of pig tissue, and it has an important impact on pig muscle growth, weight gain, and carcass quality. The weight gain rate and feed conversion rate of pigs were affected by the protein level and the ratio of essential amino acids directly. Wang et al. [14] observed that if crude protein reduction does not exceed 4%, it is essential to supplement the diet with critical amino acids, including lysine, methionine, tryptophan, and threonine. Conversely, reductions surpassing 6% necessitate the supplementation of non-essential amino acids to avert growth impairment. Thus, careful consideration of protein levels in swine diets is imperative for optimizing growth performance and product quality.

Current research has revealed that the specific protein requirements for Ningxiang pigs remain inadequately defined. This lack of clarity may be contributing to the observed slow growth rates within the 11.09% protein group, potentially attributable to an excessive reduction in protein intake, an imbalance in amino acid composition, and inefficient amino acid utilization. Protein deposition exhibits a linear relationship up to a certain threshold, beyond which it becomes contingent upon energy availability [15,16]. An overabundance of protein intake can elevate metabolic rates, resulting in increased heat production and insufficient net energy, which in turn may diminish net protein deposition and growth performance [17].

Furthermore, findings from this study indicate a linear increase in serum GSH-Px content, correlating with higher dietary protein levels, which may reflect the utilization efficiency of dietary amino acid utilization. Despite efforts to maintain a consistent amino acid pattern, discrepancies in dietary intake and utilization persist among Ningxiang pigs, impacting amino acid transformation and leading to the increase in serum GSH-Px levels. Liu et al. [18] demonstrated that a 14% protein diet reduced plasma GSH-Px activity and the GSH concentration when compared to a 20% protein diet, suggesting that low-protein diets may impair the antioxidant capacity by inhibiting both non-enzymatic and enzymatic antioxidant defense mechanisms. In the work of Wu et al. [19], it was observed that feeding diets with high protein significantly increased the enhance serum SOD content, yet exhibited no significant effects on the serum CAT, MDA, and T-AOC levels, likely due to insufficient protein nutrients hindering antioxidant enzyme production.

This study also observed a linear decrease in the redness and yellowness parameters of *Longissimus dorsi* muscle, which decreased linearly with increasing dietary protein levels. Choi et al. [20] similarly identified detrimental effects on pork quality associated with both high and low protein levels in growing/finishing pigs. Reports indicate that the L*, a*, and b* values of *Longissimus dorsi* muscle in pigs subjected to low-protein diets are elevated [20,21], attributed to an increased intramuscular fat (IMF) content [22]. Notably, our trial recorded significant increases in IMF content across both low- and high-protein diets. Previous investigations have established that implementing low-protein diets during the growth or fattening phases can augment the IMF content of pigs without concomitant increases in back fat [23,24], possibly due to the activation of lipogenic enzyme expression within muscle tissues [25], thus facilitating de novo fatty acid synthesis.

The relationship between dietary protein levels and amino acids in *Longissimus dorsi* muscle reveals significant insights into nutritional biochemistry. Our investigation illustrates that the amino acid composition follows a quadratic trend relative to the dietary protein intake. Notably, both insufficient and excessive protein levels correlate with a diminished amino acid content in the *Longissimus dorsi* muscle, aligning with previous findings that denote a positive correlation between dietary protein and muscle protein synthesis [26,27]. This observation indicates that an imbalanced amino acid supply may instigate protein turnover, striving to achieve an equilibrium within the amino acid pool of the organism. Furthermore, contrasting reports indicate that the dietary protein level for fattening pigs is reduced from 16% to 14%, and when supplemented with essential amino acids, can enhance the EAA/TAA ratio in muscle [28]. It follows that a more balanced amino acid supply facilitates a closer alignment of tissue amino acids with the ideal protein pattern [29]. The amino acid score serves as a vital metric in assessing the nutritional value of meat, where a higher score correlates with enhanced food quality [30]. In conclusion, discrepancies observed in muscle protein and total hydrolyzed amino acid contents primarily stem from imbalances in dietary amino acid composition, a finding that merits further scrutiny and may be linked to the extent of protein degradation within muscle tissues.

The production of biogenic amines in the posterior intestine is primarily attributed to the action of amino acid decarboxylase, an enzyme found in various intestinal microorganisms, including but not limited to Bacteroides, Clostridium, Bifidobacterium, Enterobacterium, and Streptococcus [31,32]. A notable observation within the scope of this study is the inverse relationship between dietary protein levels and the concentrations of putrescine and spermidine within the cecum, aligning with findings from previous research.

Particularly significant is the finding that the spermine content in the group with 11.09% crude protein (CP) was markedly elevated compared to the other experimental groups. Spermidine is recognized for its critical involvement in various physiological processes at the cellular level, particularly in modulating metabolite alterations arising from oxidative stress, which encompasses amino acid metabolism, as well as in fostering the development of the small intestine [33,34].

The concentration of biogenic amines present in the gastrointestinal tract is contingent upon the populations of bacteria responsible for their synthesis, as well as those that facilitate their absorption. Notably, both Bacteroides and Clostridium have demonstrated capabilities for the synthesis of putrescine and spermidine, both in vivo and in vitro [35]. Putrescine serves as a precursor for spermidine, which is subsequently converted into spermidine through the enzymatic action of spermidine synthase [36].

Moreover, alterations in amino acid metabolism within the organism exert a considerable influence on the biogenic amines present in the hindgut. Intestinal short-chain fatty acids (SCFAs) are typical metabolites resulting from carbohydrate fermentation. Despite the absence of significant changes in total SCFA content during this investigation, specific fatty acids, namely isobutyric acid, butyric acid, and isovaleric acid, exhibited a linear escalation corresponding with increased dietary protein levels. This observation can largely be attributed to the carbohydrate and fiber composition of the diet. Furthermore, it is acknowledged that amino acid fermentation contributes to SCFA production, albeit at a relatively diminished rate [37]. Previous studies have reported that an elevation in dietary protein correlates with heightened concentrations of branched-chain fatty acids (BCFAs) and SCFAs in digestive samples collected from the cecum and colon, as well as in the feces of growing pigs [38,39]. Consequently, it is evident that dietary protein levels significantly influence the production of microbial metabolites within the hindgut.

The manipulation of dietary protein levels significantly influences the structure of cecal microbiota during the fattening phase of Ningxiang pigs. Notably, the Chao and Shannon indices—indicators of microbial abundance and diversity—demonstrated that the cecal floral abundance in the 11.09% dietary protein group was markedly diminished. Conversely, both the 12.09% and 13.09% protein groups exhibited a significant reduction in microbial diversity. While increased microbial diversity typically correlates with enhanced microbiotal stability and pathogen resistance, the current findings suggest that reduced diversity in the latter groups does not necessarily imply increased susceptibility to pathogen invasion [40]. At both the phylum and genus levels, the relative abundance of Firmicutes and Lactobacillus was elevated in the higher protein groups compared to the other dietary conditions. A heightened Firmicutes to Bacteroides ratio is often indicative of obesity-related metabolic disorders [41,42]. Lactobacillus, recognized for its beneficial roles in digestive health, serves to regulate gut microbiota, suppress pathogenic bacteria, and bolster intestinal immunity [43,44,45]. Moreover, a notable increase in Limosilactobacillus abundance was observed in groups with lower protein levels. Although the overall abundance of Lactobacillus in the 11.09% and 15.09% dietary protein groups declined, the presence of beneficial Lactobacillus-related genera, such as Limosilactobacillus and Ligilactobacillus, increased, suggesting a potential advantage in optimizing protein utilization in Ningxiang pigs.

## 5. Conclusions

The increase in dietary protein levels correlates with the growth performance and amino acid profile of the *Longissimus dorsi* muscle in Ningxiang pigs, presenting a quadratic relationship. Concurrently, the serum antioxidant capacity and cecal bioamine content demonstrate a linear increase. Despite a balanced inclusion of six essential amino acids, both excessively high and low protein levels adversely affect growth and intestinal health. Notably, dietary protein levels of 12.09% and 13.09% yield the optimal growth performance under the specified experimental conditions.

## Figures and Tables

**Table 1:** Ingredient composition and nutrient levels of the experimental diets (%, as-fed basis).

Item	Dietary Protein Content, %				
	11.09	12.09	13.09	14.09	15.09
Corn	65.67	64.14	62.37	60.53	58.96
Soybean meal	1.50	5.30	9.00	12.70	16.40
Rice bran meal	28.00	26.10	24.50	23.00	21.20
L-lysine-HCL	0.44	0.33	0.22	0.11	0.00
Mold inhibitor L-threonine	0.21	0.15	0.10	0.05	0.00
L-tryptophan	0.06	0.04	0.03	0.01	0.00
DL-methionine	0.06	0.04	0.03	0.01	0.00
L-valine	0.23	0.17	0.11	0.05	0.00
Isoleucine	0.23	0.17	0.11	0.05	0.00
Preventive mildew	0.05	0.05	0.05	0.05	0.05
Calcium hydrogen phosphate	0.05	0.05	0.05	0.04	0.03
Stone powder	1.20	1.16	1.13	1.10	1.06
Salt	0.30	0.30	0.30	0.30	0.30
Premix ^a^	2.00	2.00	2.00	2.00	2.00
Total	100.00	100.00	100.00	100.00	100.00
Nutrient level ^b^					
NE, MJ/kg	9.86	9.86	9.86	9.85	9.85
Crude protein	11.09	12.09	13.09	14.09	15.09
Crude protein (analyzed value)	11.01	11.98	13.02	14.03	14.99
SID lysine	0.62	0.62	0.62	0.62	0.62
SID threonine	0.46	0.46	0.46	0.46	0.46
SID tryptophan	0.12	0.12	0.12	0.12	0.12
SID methionine	0.24	0.24	0.24	0.24	0.24
SID methionine + cystine	0.42	0.43	0.45	0.45	0.47
SID valine	0.61	0.61	0.61	0.61	0.61
SID Isoleucine	0.46	0.46	0.46	0.46	0.46
Calcium	0.49	0.49	0.49	0.49	0.49
Phytate phosphorus	0.20	0.20	0.20	0.20	0.20

^a^ Supplied per kg of diet: vitamin A 18,000 IU, vitamin D 35,000 IU, vitamin E 35 IU, vitamin K 5 mg, vitamin B1 5 mg, vitamin B2 10 mg, vitamin B12 35 μg, iron 66 mg, copper 6 mg, zinc 54 mg, magnesium 15 mg, iodine 0.24 mg, selenium 0.18 mg, niacin 40 mg, pantothenic acid 20 mg, folic acid 1.5 mg. ^b^ Calculated values. The standardized ileal digestible (SID) concentrations were determined by multiplying the determined AA content in ingredients by the SID coefficients of the corresponding AA in those feedstuffs from NRC (2012) and summing the values.

**Table 2:** Fatty acid composition of the diet.

Item	Dietary Protein Content, %				
	11.09	12.09	13.09	14.09	15.09
C14:0	0.13	0.12	0.13	0.12	0.10
C15:0	0.04	0.04	0.01	0.03	0.03
C16:0	16.47	16.22	16.38	16.18	16.20
C16:1	0.21	0.24	0.19	0.25	0.26
C17:0	0.08	0.09	0.10	0.12	0.12
C18:0	2.02	2.05	2.08	2.10	2.27
C18:1n9c	23.60	23.73	23.05	23.14	22.91
C18:2n6c	54.16	53.97	54.35	54.51	54.27
C18:3n3	1.60	1.79	2.09	1.98	2.24
C20:0	0.44	0.45	0.44	0.44	0.44
C20:1	0.30	0.30	0.27	0.29	0.26
C21:0	0.05	0.04	0.04	0.04	0.04
C22:0	0.20	0.23	0.24	0.22	0.25
C22:1n9	0.14	0.07	0.09	0.07	0.05

**Table 3:** Effects of different dietary protein levels on the growth performance of Ningxiang pigs.

Item ^1^	Dietary Protein Content, %					SEM ^2^	*p*-Value		
	11.09	12.09	13.09	14.09	15.09		ANOVA	Linear	Quadratic
Initial BW, kg	53.39	53.06	53.06	53.26	53.18	1.06	0.99	0.94	0.86
final BW, kg	79.06 ^b^	81.72 ^a^	81.34 ^a^	80.13 ^ab^	78.55 ^b^	0.66	0.01	0.25	<0.01
ADG, g	421.12 ^bc^	469.9 ^a^	463.66 ^ab^	443.69 ^abc^	415.95 ^c^	13.90	0.04	0.42	<0.01
ADFI, g	1937.38	2012.23	2015.95	1956.77	1952.18	39.20	0.50	0.84	0.15
F/G	4.60 ^ab^	4.29 ^c^	4.36 ^bc^	4.42 ^bc^	4.70 ^a^	0.08	0.01	0.23	<0.01

^1^ BW, body weight; ADG, average daily gain; ADFI, average daily feed intake; and F/G, feed/gain ratio. ^2^ SEM standard error of the means. Data are the means of five replicates of five pigs per pen. ^a, b, c^, Different superscripts within a row indicate a significant difference (*p* < 0.05).

**Table 4:** Effects of different dietary protein levels on carcass and meat quality traits of Ningxiang pigs.

Item ^1^	Dietary Protein Content, %					SEM ^2^	*p*-Value		
	11.09	12.09	13.09	14.09	15.09		ANOVA	Linear	Quadratic
Carcass traits									
Carcass weight, kg	59.55	60.29	59.49	57.93	56.47	1.16	0.19	0.03	0.25
Eye muscle area, cm^2^	26.76	29.58	30.59	27.28	32.95	2.91	0.57	0.29	0.89
Thickness of backfat, mm	38.99	40.36	35.82	38.78	36.58	2.32	0.64	0.39	0.97
Meat percentage, %	38.96	40.68	42.89	39.72	41.62	1.11	0.15	0.23	0.24
Carcass straight length, cm	86.00	88.00	86.30	85.10	87.20	1.40	0.64	0.91	0.89
Carcass skew length, cm	76.10	75.00	77.10	75.60	75.70	1.29	0.83	0.96	0.81
Meat quality traits									
Muscle color									
L*	44.34	45.94	45.04	44.34	45.52	0.97	0.71	0.81	0.87
a*	10.36 ^a^	10.06 ^a^	8.86 ^b^	8.84 ^b^	8.61 ^b^	0.37	0.01	<0.01	0.36
b*	7.85 ^ab^	8.10 ^a^	6.93 ^b^	7.13 ^b^	6.93 ^b^	0.29	0.03	<0.01	0.67
Muscle pH									
pH_45min_	6.65	6.46	6.31	6.51	6.35	0.11	0.26	0.14	0.33
pH_24h_	5.52	5.66	5.50	5.70	5.63	0.08	0.37	0.35	0.87
Drip loss at 24 h, %	2.09	1.62	1.70	1.92	2.21	0.32	0.65	0.61	0.18
Intramuscular fat, %	3.63 ^a^	2.97 ^ab^	2.64 ^b^	2.92 ^ab^	3.48 ^a^	0.23	0.04	0.63	<0.01
Water-holding capacity, %	16.42	16.52	22.38	17.83	22.28	2.06	0.12	0.06	0.83
Shear force, %	39.30	46.83	48.05	42.40	43.65	6.74	0.89	0.84	0.45

^1^ For L*, lightness; a*, redness; and b*, yellowness. ^2^ SEM standard error of the means. Data are the means of five replicates of five pigs per pen. ^a, b^, different superscripts within a row indicate a significant difference (*p* < 0.05).

**Table 5:** Effects of different dietary protein levels on fatty acid profiles of the *Longissimus dorsi* muscle of Ningxiang pigs (% of total fatty acids).

Item ^1^	Dietary Protein Content, %					SEM ^2^	*p*-Value		
	11.09	12.09	13.09	14.09	15.09		ANOVA	Linear	Quadratic
C14:0	1.19	1.31	1.18	1.26	1.33	0.04	0.08	0.09	0.56
C14:1	0.03	0.03	0.03	0.04	0.03	0.003	0.36	0.58	0.10
C16:0	25.35	26.04	25.49	25.82	25.66	0.38	0.74	0.75	0.58
C16:1	3.39	3.63	3.08	3.60	3.21	0.22	0.34	0.59	0.81
C17:0	0.13 ^c^	0.13 ^bc^	0.14 ^abc^	0.15 ^ab^	0.16 ^a^	0.008	0.04	<0.01	0.69
C17:1	0.08 ^b^	0.09 ^b^	0.08 ^b^	0.09 ^b^	0.11 ^a^	0.006	0.02	0.01	0.08
C18:0	13.41	13.34	13.59	13.73	13.54	0.38	0.95	0.59	0.81
C18:1n9t	0.07	0.05	0.08	0.07	0.07	0.008	0.11	0.18	0.90
C18:1n9c	41.82	41.46	39.41	41.40	41.67	0.81	0.25	0.89	0.10
C18:2n6c	9.26	9.19	10.59	9.89	9.16	0.62	0.46	0.96	0.21
C18:3n3	0.25 ^b^	0.24 ^b^	0.28 ^ab^	0.27 ^ab^	0.32 ^a^	0.02	0.03	<0.01	0.26
C18:3n6	0.05 ^b^	0.06 ^ab^	0.08 ^ab^	0.09 ^a^	0.07 ^ab^	0.01	0.09	0.03	0.12
C20:0	0.26	0.24	0.25	0.24	0.36	0.05	0.43	0.24	0.19
C20:1	0.79	0.72	0.86	0.71	0.67	0.08	0.46	0.34	0.42
C20:2	0.29	0.27	0.35	0.33	0.34	0.03	0.17	0.06	063
C20:3n6	0.27	0.29	0.38	0.41	0.26	0.05	0.10	0.43	0.03
C20:3n3	0.05	0.05	0.05	0.05	0.06	0.01	0.41	0.13	0.27
C20:4n6	2.15	2.25	2.48	2.45	1.66	0.27	0.26	0.38	0.06
SFA	40.42	41.15	40.74	41.29	41.13	0.61	0.84	0.43	0.73
MUFA	46.17	45.98	43.55	45.90	45.77	0.91	0.27	0.77	0.17
PUFA	12.35	12.39	14.27	13.73	11.92	0.99	0.42	0.88	0.12
MUFA/PUFA	3.81	3.76	3.11	3.51	3.90	0.31	0.41	0.93	0.12

^1^ SFA = saturated fatty acids; MUFA = monounsaturated fatty acids; PUFA = polyunsaturated fatty acids; ^2^ SEM standard error of the means. Data are the means of five replicates of five pigs per pen. ^a, b, c^, Different superscripts within a row indicate a significant difference (*p* < 0.05).

**Table 6:** Effects of different dietary protein levels on amino acids of the *Longissimus dorsi* muscle of Ningxiang pigs (g/100g).

Item ^1^	Dietary Protein Content, %					SEM ^2^	*p*-Value		
	11.09	12.09	13.09	14.09	15.09		ANOVA	Linear	Quadratic
Asp	5.49	5.61	5.97	5.92	5.49	0.18	0.20	0.59	0.04
Thr	2.63 ^b^	2.86 ^ab^	3.07 ^a^	3.05 ^a^	2.81 ^ab^	0.08	0.01	0.06	<0.01
Ser	2.38	2.37	2.54	2.53	2.32	0.08	0.26	0.89	0.08
Glu	7.67 ^b^	8.02 ^ab^	8.39 ^a^	8.15 ^a^	8.01 ^ab^	0.14	0.04	0.09	0.01
Gly	2.40 ^b^	2.61 ^ab^	2.84 ^a^	2.81 ^a^	2.51 ^b^	0.07	<0.01	0.09	<0.01
Ala	3.19 ^b^	3.51 ^ab^	3.78 ^a^	3.77 ^a^	3.42 ^b^	0.11	<0.01	0.05	<0.01
Cys	0.35	0.36	0.38	0.39	0.35	0.01	0.22	0.49	0.05
Val	2.85 ^b^	3.13 ^ab^	3.32 ^a^	3.33 ^a^	3.04 ^ab^	0.09	0.01	0.07	<0.01
Met	1.44	1.59	1.64	1.63	1.55	0.07	0.32	0.27	0.07
Ile	2.96	2.96	3.15	3.16	2.89	0.11	0.36	0.85	0.10
Leu	4.92 ^b^	5.18 ^ab^	5.54 ^a^	5.56 ^a^	5.04 ^b^	0.15	0.03	0.22	<0.01
Tyr	2.17 ^b^	2.24 ^ab^	2.36 ^a^	2.38 ^a^	2.17 ^b^	0.06	0.04	0.49	<0.01
Phe	2.42 ^b^	2.58 ^ab^	2.75 ^a^	2.77 ^a^	2.50 ^b^	0.07	0.01	0.16	<0.01
Lys	2.31	2.25	2.35	2.34	2.20	0.06	0.37	0.86	0.09
NH3	0.84	0.85	0.90	0.89	0.83	0.03	0.17	0.41	0.03
His	2.42 ^b^	2.70 ^ab^	2.84 ^a^	2.89 ^a^	2.65 ^ab^	0.09	0.02	0.03	<0.01
Arg	3.66 ^b^	3.87 ^ab^	4.17 ^a^	4.15 ^a^	3.77 ^b^	0.11	0.02	0.15	<0.01
Pro	2.32 ^b^	2.38 ^b^	2.60 ^a^	2.57 ^a^	2.33 ^b^	0.06	<0.01	0.25	<0.01
EAA	25.52 ^b^	27.12 ^ab^	28.84 ^a^	28.88 ^a^	26.46 ^ab^	0.78	0.03	0.16	<0.01
NEAA	25.98 ^b^	27.11 ^ab^	28.86 ^a^	28.52 ^a^	26.61 ^b^	0.57	0.01	0.14	<0.01
Total AA	52.34 ^b^	55.08 ^ab^	58.60 ^a^	58.30 ^a^	53.89 ^b^	1.36	0.02	0.16	<0.01

^1^ EAA = essential amino acid; NEAA = non-essential amino acid; Total AA = total amino acid. ^2^ SEM standard error of the means. Data are the means of five replicates of five pigs per pen. ^a, b^, Different superscripts within a row indicate a significant difference (*p* < 0.05).

**Table 7:** Effects of different dietary protein levels on the serum antioxidant capacity of Ningxiang pigs.

Item ^1^	Dietary Protein Content, %					SEM ^2^	*p*-Value		
	11.09	12.09	13.09	14.09	15.09		ANOVA	Linear	Quadratic
SOD (U/mL)	86.60	90.22	85.77	87.55	87.05	1.89	0.54	0.77	0.78
MDA (nmol/mL)	2.83	2.98	3.02	2.97	2.85	0.08	0.32	0.93	0.06
GSH-Px (U/mL)	382.05 ^b^	403.95 ^a^	419.96 ^a^	426.11 ^a^	418.38 ^a^	7.18	<0.01	<0.01	0.02
T-AOC (U/mL)	3.78	3.70	3.75	3.88	3.76	0.07	0.44	0.53	0.95

^1^ SOD = superoxide dismutase; MDA = malondialdehyde; GSH-Px = glutathione peroxidase; T-AOC = total antioxidant capacity. ^2^ SEM standard error of the means. Data are the means of five replicates of five pigs per pen. ^a, b^, Different superscripts within a row indicate a significant difference (*p* < 0.05).

**Table 8:** Effects of different dietary protein levels on cecal biogenic amine contents of Ningxiang pigs (μg/g).

Item	Dietary Protein Content, %					SEM ^1^	*p*-Value		
	11.09	12.09	13.09	14.09	15.09		ANOVA	Linear	Quadratic
Tyramine	0.82	0.81	0.82	0.82	0.81	0.01	0.31	0.24	0.51
Putrescine	21.72 ^b^	22.31 ^b^	22.85 ^ab^	23.78 ^a^	23.65 ^a^	0.36	0.02	<0.01	0..47
Cadaverine	1.13 ^e^	2.46 ^d^	6.07 ^c^	12.88 ^b^	23.87 ^a^	0.05	<0.01	<0.01	<0.01
Spermidine	13.75 ^c^	18.98 ^b^	23.56 ^ab^	32.30 ^a^	36.33 ^a^	1.57	<0.01	<0.01	0.77
Spermine	10.65 ^a^	4.15 ^b^	4.55 ^b^	4.64 ^b^	4.11 ^b^	0.20	<0.01	<0.01	<0.01
Tryptamine	1.26	1.24	1.16	1.32	1.20	0.05	0.27	0.79	0.89
Histamine	8.23	8.26	8.25	8.37	8.52	0.16	0.67	0.21	0.53

^1^ SEM standard error of the means. Data are the means of five replicates of five pigs per pen. ^a, b, c, d, e^, Different superscripts within a row indicate a significant difference (*p* < 0.05).

**Table 9:** Effects of different dietary protein levels on colonic SCFA contents of Ningxiang pigs (μg/mL).

Item ^1^	Dietary Protein Content, %					SEM ^2^	*p*-Value		
	11.09	12.09	13.09	14.09	15.09		ANOVA	Linear	Quadratic
Acetic acid	254.09	165.48	261.30	201.52	224.52	28.52	0.17	0.80	0.54
Propionic acid	114.71	86.62	105.55	115.06	129.11	13.66	0.32	0.21	0.17
Isobutyric acid	6.00 ^b^	5.32 ^b^	7.76 ^b^	12.27 ^a^	8.79 ^ab^	1.19	0.01	<0.01	0.44
Butyric acid	53.98 ^ab^	31.42 ^c^	39.01 ^bc^	61.94 ^a^	56.92 ^ab^	6.92	0.04	0.12	0.08
Isovaleric acid	9.66 ^b^	8.56 ^b^	11.95 ^b^	22.85 ^a^	15.35 ^b^	2.31	<0.01	<0.01	0.55
Valeric acid	10.26	9.36	10.07	12.62	11.50	1.42	0.53	0.23	0.80
Total SCFA	448.70	316.77	435.64	426.26	446.19	42.80	0.22	0.46	0.29

^1^ Total SCFA = acetic acid + propionic acid + isobutyric acid + butyric acid + isovaleric acid + valeric acid. ^2^ SEM standard error of the means. Data are the means of five replicates of five pigs per pen. ^a, b, c^, Different superscripts within a row indicate a significant difference (*p* < 0.05).

## Data Availability

None of the data were deposited in an official repository. Data that support the study findings are available upon request.
